# Risk Factors for Anastomotic Stricture and Obstructive Symptoms Following Double‐Flap Technique Reconstruction After Proximal Gastrectomy

**DOI:** 10.1002/ags3.70130

**Published:** 2025-11-21

**Authors:** Shinji Kuroda, Yoshihiko Kakiuchi, Satoru Kikuchi, Hajime Kashima, Nobuhiko Kanaya, Shunya Hanzawa, Kenjiro Kumano, Masahiko Nishizaki, Shunsuke Kagawa, Toshiyoshi Fujiwara

**Affiliations:** ^1^ Department of Gastroenterological Surgery Okayama University Graduate School of Medicine, Dentistry and Pharmaceutical Sciences Okayama Japan; ^2^ Department of Surgery Japanese Red Cross Okayama Hospital Okayama Japan; ^3^ Department of Surgery Tsuyama Chuo Hospital Tsuyama Japan

**Keywords:** anastomotic stricture, double‐flap technique, Kamikawa procedure, obstructive symptoms, proximal gastrectomy

## Abstract

**Background:**

The double‐flap technique (DFT) is a widely used esophagogastrostomy method after proximal gastrectomy (PG) due to its strong anti‐reflux mechanism. However, anastomotic stricture remains a major concern, leading to obstructive symptoms and impaired postoperative quality of life (QOL). This study aimed to identify risk factors for anastomotic stricture and obstructive symptoms after PG with DFT reconstruction.

**Methods:**

This single‐center, retrospective study analyzed 77 patients who underwent DFT reconstruction between 2014 and 2022. The impact of technical factors, including suturing methods and mucosal detachment, was evaluated. In addition, intraluminal pressure analysis was performed using ex vivo pig stomach models to assess sites contributing to obstructive symptoms.

**Results:**

Anastomotic stricture requiring balloon dilatation occurred in 10 patients (13%), with mucosal detachment of the esophagus identified as an independent risk factor (odds ratio [OR]: 48, 95% confidence interval [CI]: 4.47–515, *p* = 0.001). Thermal damage during esophageal transection was a potential risk factor for mucosal detachment (OR: 6.63, 95% CI: 1.00–44.1, *p* = 0.051). Moderate or severe obstructive symptoms 1 month after surgery were reported by 59% of patients, with continuous suturing for esophago‐stomach fixation (E‐S fixation) increasing the risk (OR: 4.50, 95% CI: 0.98–20.7, *p* = 0.054). Intraluminal pressure analysis confirmed that continuous suturing at the E‐S fixation site significantly increased pressure compared with interrupted suturing (*p* = 0.028).

**Conclusion:**

Preventing mucosal detachment by minimizing thermal damage can reduce anastomotic stricture. Further, interrupted suturing at the E‐S fixation site may reduce obstructive symptoms. These findings provide insights into optimizing DFT reconstruction to improve postoperative outcomes and patient QOL.

## Graphical abstract

This retrospective study investigated risk factors for anastomotic stricture and obstructive symptoms following double‐flap technique (DFT) reconstruction after proximal gastrectomy. Mucosal detachment, potentially caused by thermal damage, was identified as a significant risk factor for anastomotic stricture, while continuous suturing at the esophago‐stomach fixation site increased intraluminal pressure and obstructive symptoms. Minimizing thermal injury and using interrupted sutures may optimize DFT outcomes and improve postoperative quality of life.

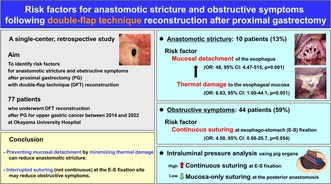

## 1 Introduction

Double‐flap technique (DFT) is a method of esophagogastrostomy (EG) after proximal gastrectomy (PG), originally developed by Dr. Kamikawa and first reported in 1998. This technique is primarily performed by a completely hand‐sewn suturing process and is characterized by its strong anti‐reflux mechanism, which is achieved by creating a one‐way valve with the distal esophagus [1]. By utilizing the gastric wall to create a double‐layered flap, DFT effectively prevents gastroesophageal reflux, addressing one of the major challenges associated with PG. To evaluate the clinical outcomes of this technique, we previously conducted a multi‐center, retrospective study (rD‐FLAP Study), in which the incidence of reflux esophagitis of all grades according to the Los Angeles (LA) classification 1 year after surgery was 10.6%, and that of LA ≥ B was 6.0% [2]. Furthermore, we have recently conducted a multi‐center, prospective study (lD‐FLAP study) to show the efficacy of laparoscopic DFT, in which the incidence of reflux esophagitis of LA all grades was 13.2%, and that of LA ≥ B was 5.3% [3]. Many other studies have reported the efficacy of DFT reconstruction for its anti‐reflux potential. Based on these findings, DFT is recognized as one of the representative reconstruction procedures after PG, along with modified side overlap esophagogastrostomy (mSOFY) and double‐tract reconstruction [4, 5].

However, the most critical complication after DFT reconstruction is anastomotic stricture, which can lead to obstructive symptoms in the epigastric region, impaired oral intake, and reduced quality of life for patients postoperatively. In our previous studies, the incidence of anastomotic stricture requiring endoscopic balloon dilatation was 5.5% in the rD‐FLAP study and 5.3% in the lD‐FLAP study [2, 3]. In other studies, its incidence has been reported to range from 8.3% to 18% [6, 7, 8, 9, 10]. In general, various factors, including technical aspects of the anastomosis, postoperative complications such as anastomotic leakage, preoperative treatment such as radiotherapy, and patient‐related factors such as smoking, are considered risk factors contributing to anastomotic stricture in gastrointestinal surgery [11, 12, 13, 14]. Specifically in DFT reconstruction, diameter of the esophagus < 18 mm evaluated on pre‐operative computed tomography (CT) was reported as an independent risk factor for anastomotic stricture after laparoscopic DFT, along with the presence of short‐term complications [6]. In another report, an excessive number of stiches on the anastomosis was reported as a significant risk factor after robotic DFT reconstruction [15].

In this single‐center, retrospective study, the aim was to investigate the risk factors for anastomotic stricture and obstructive symptoms after PG, with a particular focus on the technical aspects of DFT reconstruction. Specifically, the impact of different suturing techniques, interrupted versus continuous suturing and full‐thickness versus mucosal suturing, as well as the presence or absence of mucosal detachment of the esophagus at the anastomotic site, was examined. Furthermore, intraluminal pressure in the reconstructed area was measured using harvested pig stomachs ex vivo to identify the frequent sites and risk factors for obstructive symptoms after PG. These findings may provide valuable insights into optimizing DFT reconstruction procedures to reduce the incidence of anastomotic stricture and obstructive symptoms.

## 2 Methods

### 2.1 Study Design and Patients

This was designed as a single‐center, single‐arm, retrospective study. This study conformed to the provisions of the Declaration of Helsinki, and the protocol was approved by the Okayama University Hospital Institutional Review Board (Approval no. 2504‐027).

Patients who underwent DFT reconstruction after PG for GC located in the upper third of the stomach at Okayama University Hospital between June 2014 and June 2022 were enrolled. Patients diagnosed with esophagogastric junction (EGJ) cancer or those who underwent this procedure for other diseases, such as submucosal tumors, were excluded.

### 2.2 DFT Procedure and Modifications

**FIGURE 1:**
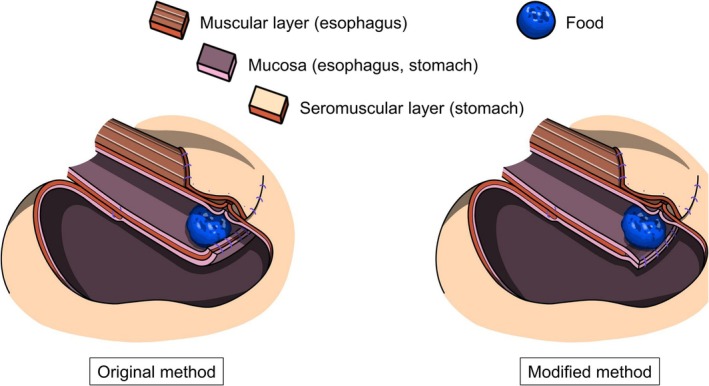
Schematic illustration of the modification from full‐thickness suturing to mucosal suturing of the esophagus on the posterior anastomosis.

The fundamental step‐by‐step procedure and technique of DFT have been described in previous reports [1, 16]. Throughout the study period, several minor technical modifications aimed at simplifying the procedure and reducing the incidence of anastomotic stricture and obstructive symptoms were made. These modifications included: (1) using either interrupted or continuous suturing for fixation of the esophagus and the remnant stomach (E‐S fixation); (2) performing either full‐thickness or mucosal suturing of the esophagus on the posterior side of the anastomosis; and (3) using either interrupted or continuous suturing for the second‐layer suturing on the anterior side of the anastomosis. Regarding the second modification, the suturing procedure for the esophagus on the posterior side of the anastomosis was changed from full‐thickness suturing (the original method) to mucosal suturing, based on the concept that this would increase flexibility of the anastomosis, thus preventing obstructive symptoms, without increasing the risk of anastomotic leakage (Figure 1).

### 2.3 Data Collection

**FIGURE 2:**
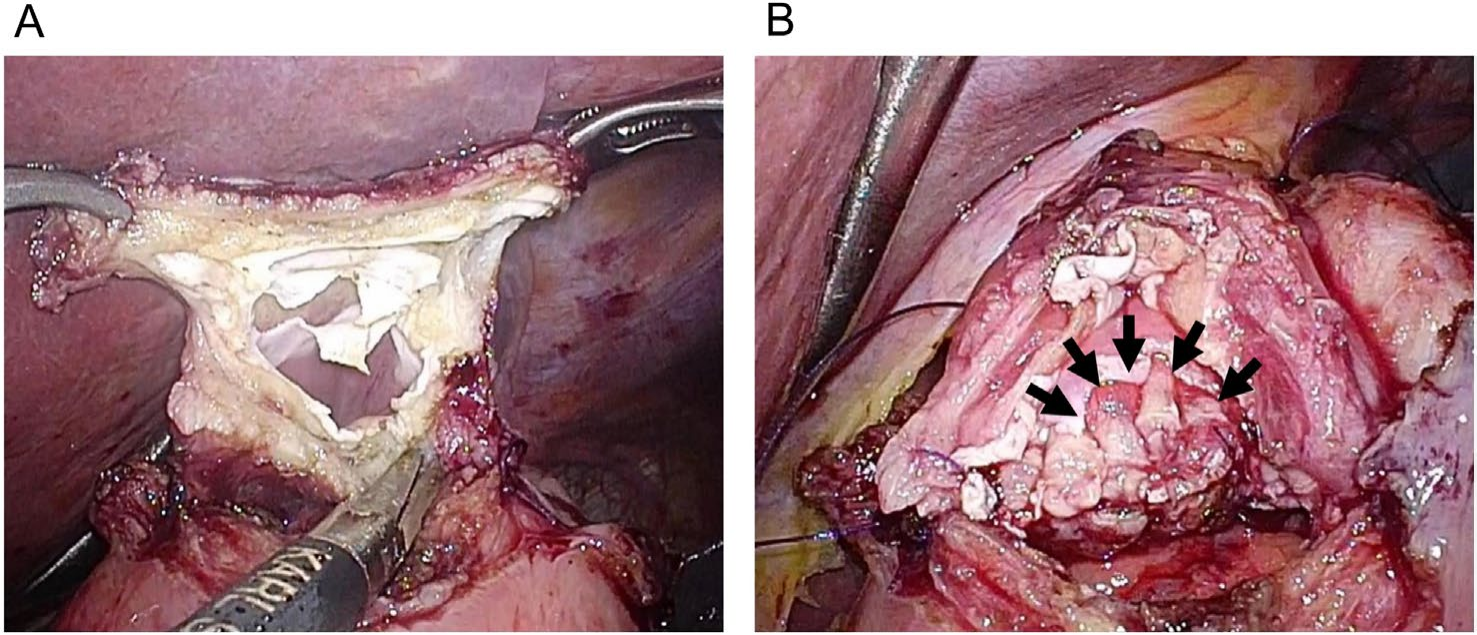
Representative images of thermal damage and mucosal detachment from the same case. (A) Thermal damage of the esophageal mucosa during transection of the esophageal staple line. (B) Image at completion of the posterior wall anastomosis. Note that the arrows indicate the area of mucosal detachment of the esophagus.

Data on patients' background characteristics, including age, sex, body mass index (BMI; weight [kg]/height [m]^2^), and prognostic nutritional index (PNI; 10 × serum albumin [g/dL] + 0.005 × total lymphocyte count [/mm^3^]), as well as surgical factors such as approach (open, laparoscopy, robot), operation time, and blood loss, were obtained from the medical records. Surgical outcomes, including postoperative complications during the first hospitalization, recorded according to the Clavien–Dindo (CD) classification [17], postoperative length of hospital stay, anastomotic stricture, reflux esophagitis, and obstructive symptoms, were also collected. Obstructive symptoms were classified into four categories: none (no obstructive symptoms); mild (symptoms mostly not requiring interruption of oral intake); moderate (symptoms requiring interruption of oral intake); and severe (symptoms accompanied by occasional vomiting). The diameter of the esophagus was measured at the level of the crura of the diaphragm on preoperative CT, as described in a previous report [6]. Information regarding the details of the DFT procedure described above, thermal damage to the esophageal mucosa during esophageal transection (Figure 2A), energy device used for esophageal transection, and mucosal detachment of the esophagus during anastomosis (Figure 2B) was obtained from the medical records or surgical video. Mucosal detachment of the esophagus was defined as present when the mucosal anastomosis was insufficient for more than one‐quarter of the circumference.

### 2.4 Intraluminal Pressure Analysis

Using the distal esophagus and the stomach of a pig, kindly provided by ETHICON, a part of Johnson & Johnson MedTech (Tokyo, Japan), DFT reconstruction was performed ex vivo by two different procedures (*n* = 3). One was the previous procedure, in which E‐S fixation was done with continuous suturing, and the suturing of the esophagus on the posterior side of the anastomosis was done with full‐thickness suturing, and the other was the current procedure, in which the former was done with interrupted suturing and the latter with mucosal suturing. All reconstruction steps for both procedures were performed by a single surgeon to minimize potential technical bias. Intraluminal pressure (kPa) was measured at the following sites: (1) the proximal site of the anastomosis (baseline); (2) the E‐S fixation site; (3) the intra‐flap area; and (4) the anastomotic site. A urethral catheter with a balloon moderately dilated by water, connected to a pressure‐measuring device, was used to measure the pressure (Video S1). The differences in pressure between (2), (3), and (4) versus (1) were then calculated.

### 2.5 Statistical Analysis

Statistical analyses were performed using Stata/SE version 19.5 (StataCorp LLC, College Station, TX, USA). Continuous variables such as age, BMI, PNI, diameter of the esophagus, and the difference in intraluminal pressure are expressed as average ± standard deviation (SD) values, and continuous variables such as operation time, blood loss, and postoperative length of hospital stay are expressed as median (range) values. Student's *t*‐test was used to compare age, BMI, PNI, and the diameter of the esophagus between two groups after confirmation of normality, and the Mann–Whitney *U* test was used to compare non‐parametric variables such as operation time and blood loss. Categorical variables such as sex, surgical approach, postoperative complications, details of the DFT procedure, the presence of mucosal detachment, thermal damage, and type of energy device were compared using Pearson's chi‐squared test. The correlation coefficient (*r*) was used to assess the relationship between anastomotic stricture onset and the number of dilatations required. Multivariate analysis was performed using logistic regression to evaluate risk factors for anastomotic stricture, mucosal detachment, thermal damage, and obstructive symptoms. A *p* value < 0.05 was considered significant.

## 3 Results

A total of 77 patients were enrolled in this study, and five surgeons served as operating surgeons. Table 1 presents the details of the patients' characteristics. The average age was 71.0 years, with a male‐to‐female ratio of 56 to 21. The average BMI was 23.6 kg/m^2^, and the average PNI was 50.5. The surgical approach was laparoscopic in 70 cases and robotic in 7 cases, with no open surgeries performed. The median operation time was 327 min, and the median blood loss was 80 mL. Postoperative complications (CD any grade) occurred in 10 patients (13%) during the first hospitalization, including pancreatic fistula in 1 patient (1%), abdominal abscess in 3 patients (4%), ileus in 2 patients (3%), urinary tract infection in 3 patients (4%), and other complications in 2 patients (3%). No cases of anastomotic leakage were observed. CD grade III or higher postoperative complications were observed in 3 patients (4%), including ileus in one, hiatal hernia in one, and both pancreatic fistula and abdominal abscess in a single patient. The median postoperative length of hospital stay was 11 days. During postoperative follow‐up, 10 patients (13%) had an anastomotic stricture requiring balloon dilatation. The median postoperative time to onset was 56 days, with the earliest onset at 17 days and the latest at 430 days after surgery. The median number of balloon dilatations required was 3, with a range of 2 to 8. There was no strong correlation between anastomotic stricture onset (postoperative days until the first dilatation) and the number of dilatations required (*r* = −0.3440, *p* = 0.3304). No patient developed reflux esophagitis of LA ≥ B (0%) of the 74 patients who underwent endoscopic evaluation 1 year after surgery, including those who required balloon dilatation for anastomotic stricture.

**TABLE 1:** Patients' characteristics.

	(*n* = 77)
Background	
Age, year, average ± SD	71.0 ± 9.3
Sex, male/female	56/21 (73%/27%)
BMI, kg/m^2^, average ± SD	23.6 ± 3.4
PNI, average ± SD	50.5 ± 4.5
Surgical factors	
Approach, open/laparoscopic/robotic	0/70/7 (0%/91%/9%)
Operation time, min, median (range)	327 (221–551)
Blood loss, mL, median (range)	80 (0–1130)
Surgical outcomes (1st hospitalization)	
Postoperative complications, CD any grade	10 (13%)
Anastomotic leakage	0 (0%)
Pancreatic fistula	1 (1%)
Abdominal abscess	3 (4%)
Ileus	2 (3%)
Urinary tract infection	3 (4%)
Others	2 (3%)
Postoperative hospital stay, days, median (range)	11 (8–37)
Surgical outcomes (mid‐ and long‐term)	
Anastomotic stricture, requiring balloon dilatation	10 (13%)
Postoperative time to onset, days, median (range)	56 (17–430)
Balloon dilatation, times, median (range)	3 (2–8)
Reflux esophagitis, LA grade ≥ B	0 (0%)

Abbreviations: BMI, body mass index; CD, Clavien–Dindo classification; LA, Los Angeles classification; PNI, prognostic nutritional index; SD, standard deviation.

Table 2 presents the analysis of risk factors for anastomotic stricture. On univariate analysis, potential factors were compared between 10 cases with and 67 cases without anastomotic stricture. No significant correlations with anastomotic stricture were observed for age (*p* = 0.877), sex (*p* = 0.836), BMI (*p* = 0.128), PNI (*p* = 0.296), and postoperative complications (CD any grade) (*p* = 0.763). The average diameter of the esophagus in cases with and without anastomotic stricture was 18.8 and 19.9 mm, respectively, with no significant difference (*p* = 0.286). Regarding surgical factors, all seven cases performed with the robotic approach were without anastomotic stricture (0%), although the difference was not significant (*p* = 0.284). There were no significant differences in operation time (*p* = 0.898) and blood loss (*p* = 0.903). Regarding the technical details of the DFT procedure, E‐S fixation was performed with continuous suturing in 2 cases (20%) with anastomotic stricture and in 23 cases (34%) without anastomotic stricture, with no significant difference (*p* = 0.367). The suturing of the esophagus on the posterior side of the anastomosis was done with mucosal suturing, not the original full‐thickness suturing, in no cases (0%) with anastomotic stricture and in 12 cases (18%) without anastomotic stricture, showing a moderate tendency, but no significant difference (*p* = 0.145). The second‐layer suturing on the anterior side of the anastomosis was done with continuous suturing, not interrupted suturing, in 3 cases (30%) with anastomotic stricture and in 37 cases (55%) without anastomotic stricture, showing a moderate tendency, but no significant difference (*p* = 0.136). Mucosal detachment of the esophagus was observed in 4 cases (40%) with anastomotic stricture and in 1 case (2%) without anastomotic stricture, with a significant difference (*p* < 0.001). Thermal damage to the esophageal mucosa, observed in 15 cases and mainly caused by an energy device during transection of the esophageal staple line, occurred in 3 cases (30%) with anastomotic stricture and in 12 cases (18%) without anastomotic stricture, with no significant difference (*p* = 0.351). The type of energy device used for transection of the esophageal staple line was monopolar scissors (MS) in 60 cases and an ultrasonically activated device (USAD) in 17 cases. MS were used in 7 cases (70%) with anastomotic stricture and in 53 cases (79%) without anastomotic stricture, with no significant difference (*p* = 0.517). The multivariate analysis identified mucosal detachment of the esophagus as an independent risk factor for anastomotic stricture (odds ratio [OR]: 48, 95% confidence interval [CI]: 4.47–515, *p* = 0.001).

**TABLE 2:** Risk factors for anastomotic stricture.

	Univariate analysis			Multivariate analysis		
	Stricture (*n* = 10)	No stricture (*n* = 67)	*p*	Odds ratio	95% CI	*p*
Patient factors						
Age, year, average ± SD	70.6 ± 9.7	71.1 ± 9.3	0.877			
Sex, male	7 (70%)	49 (73%)	0.836			
Preoperative BMI, kg/m^2^, average ± SD	22.1 ± 2.3	23.8 ± 3.5	0.128			
Preoperative PNI, average ± SD	49.1 ± 3.8	50.7 ± 4.6	0.296			
Diameter of the esophagus, mm, average ± SD	18.8 ± 2.2	19.9 ± 2.9	0.286			
Surgical factors						
Approach, robotic	0 (0%)	7 (10%)	0.284			
Operation time, min, median (range)	331.5 (221–469)	326 (237–551)	0.898			
Blood loss, mL, median (range)	70 (0–250)	80 (0–1130)	0.903			
E‐S fixation, continuous	2 (20%)	23 (34%)	0.367			
Anastomosis (esophagus of the posterior side), mucosa only	0 (0%)	12 (18%)	0.145		—	
Anastomosis (second layer of the anterior side), continuous	3 (30%)	37 (55%)	0.136			
Energy device, MS	7 (70%)	53 (79%)	0.517			
Surgical outcomes						
Postoperative complications, CD any grade	1 (10%)	9 (13%)	0.763			
Mucosal detachment (esophagus)	4 (40%)	1 (2%)	< 0.001	48	4.47–515	0.001
Thermal damage	3 (30%)	12 (18%)	0.351			

Abbreviations: BMI, body mass index; CD, Clavien–Dindo classification; CI, confidence interval; E‐S fixation, fixation of the esophagus and the remnant stomach; MS, monopolar scissors; PNI, prognostic nutritional index; SD, standard deviation.

Table 3 presents the analysis of potential risk factors for mucosal detachment of the esophagus, focusing on three variables, thermal damage to the esophageal mucosa, the type of energy device used for transection of the esophageal staple line (MS or USAD), and the suturing technique on the posterior esophagus at the anastomosis (mucosa only or full‐thickness), all of which are considered closely related to mucosal detachment. Of 5 cases with mucosal detachment, thermal damage was observed in 3 cases (60%), a significantly higher incidence than in those without mucosal detachment (18%) (*p* = 0.029). MS were used in 4 cases (80%) with mucosal detachment, but this was not a significant factor (*p* = 0.817). Mucosa‐only suturing was performed in 12 cases, none of which resulted in mucosal detachment (0%), although the difference was not significant (*p* = 0.291). On multivariate analysis, thermal damage to the esophageal mucosa was identified as a potential risk factor for mucosal detachment of the esophagus (OR: 6.63, 95% CI: 1.00‐44.1, *p* = 0.051).

**TABLE 3:** Risk factors for mucosal detachment of the esophagus.

	Univariate analysis			Multivariate analysis		
	Mucosal detachment (*n* = 5)	No mucosal detachment (*n* = 65)	*p*	Odds ratio	95% CI	*p*
Thermal damage	3 (60%)	12 (18%)	0.029	6.63	1.00–44.1	0.051
Energy device, MS	4 (80%)	49 (75%)	0.817			
Anastomosis (esophagus of the posterior side), mucosa only	0 (0%)	12 (18%)	0.291			

Abbreviations: CI, confidence interval; MS, monopolar scissors.

Table 4 presents the analysis of potential risk factors for thermal damage to the esophageal mucosa, focusing on two variables, the type of energy device used for transection of the esophageal staple line (MS or USAD) and the surgical approach (laparoscopic or robotic). Of the 70 cases in which thermal damage could be evaluated, MS and USAD were used in 53 and 17 cases, respectively, and thermal damage tended to occur more frequently with MS than with USAD (*p* = 0.073). No significant difference or tendency was observed in the surgical approach (*p* = 0.627). The multivariate analysis identified MS as a potential risk factor for thermal damage to the esophageal mucosa (OR: 5.74, 95% CI: 0.70–47.4, *p* = 0.105). In the 15 cases with thermal damage, mucosa‐only suturing was performed in only 1 case (6.7%), whereas it was performed in 11 of 55 cases (20%) without thermal damage, with no significant difference (*p* = 0.225). These findings underscore the importance of minimizing thermal damage to the esophageal mucosa to prevent mucosal detachment of the esophagus during anastomosis, which is the significant risk factor for anastomotic stricture in DFT reconstruction, and to this end, USAD may be preferable for esophageal transection.

**TABLE 4:** Risk factors for thermal damage to the esophageal mucosa.

	Univariate analysis			Multivariate analysis		
	Thermal damage (*n* = 15)	No thermal damage (*n* = 55)	*p*	Odds ratio	95% CI	*p*
Energy device, MS	14 (93%)	39 (71%)	0.073	5.74	0.70–47.4	0.105
Approach, robotic	1 (7%)	6 (11%)	0.627			

Abbreviations: CI, confidence interval; MS, monopolar scissors.

Table 5 presents the analysis of risk factors for obstructive symptoms in the epigastric region 1 month after surgery. Of 74 patients in whom symptom assessment could be conducted 1 month after surgery, moderate or severe obstructive symptoms were present in 44 patients (59%), whereas no or mild obstructive symptoms were present in 30 patients (41%). On univariate analysis, moderate or severe obstructive symptoms were present significantly more often in cases in which E‐S fixation was done with continuous suturing (*p* = 0.002) and in which the second‐layer suturing on the anterior side of the anastomosis was done with continuous suturing (*p* = 0.010). A tendency was observed in cases of mucosal suturing on the posterior side of the anastomosis (*p* = 0.066) and of mucosal detachment of the esophagus (*p* = 0.073), but no difference was observed in the diameter of the esophagus (*p* = 0.717). The multivariate analysis including two factors demonstrated that continuous suturing for E‐S fixation tended to be a potential risk factor for obstructive symptoms (OR: 4.50, 95% CI: 0.98–20.7, *p* = 0.054), whereas no significant difference was observed in second‐layer suturing on the anterior side of the anastomosis (*p* = 0.561).

**TABLE 5:** Risk factors for obstructive symptoms in the epigastric region 1 month after surgery.

	Univariate analysis			Multivariate analysis		
	Moderate, severe (*n* = 44)	None, mild (*n* = 30)	*p*	Odds ratio	95% CI	*p*
Patient factors						
Preoperative PNI, average ± SD	50.2 ± 4.0	51.2 ± 5.1	0.353			
Diameter of the esophagus, mm, average ± SD	19.8 ± 3.0	19.6 ± 2.7	0.717			
Surgical factors						
Approach, robotic	4 (9%)	3 (10%)	0.896			
E‐S fixation, continuous	21 (48%)	4 (13%)	0.002	4.50	0.98–20.7	0.054
Anastomosis (esophagus of the posterior side), mucosa only	10 (23%)	2 (7%)	0.066			
Anastomosis (second layer of the anterior side), continuous	28 (64%)	10 (33%)	0.010	1.46	0.41–5.21	0.561
Surgical outcome						
Mucosal detachment (esophagus)	5 (12%)	0 (0%)	0.073			

Abbreviations: CI, confidence interval; E‐S fixation, fixation of the esophagus and the remnant stomach; PNI, prognostic nutritional index; SD, standard deviation.

**FIGURE 3:**
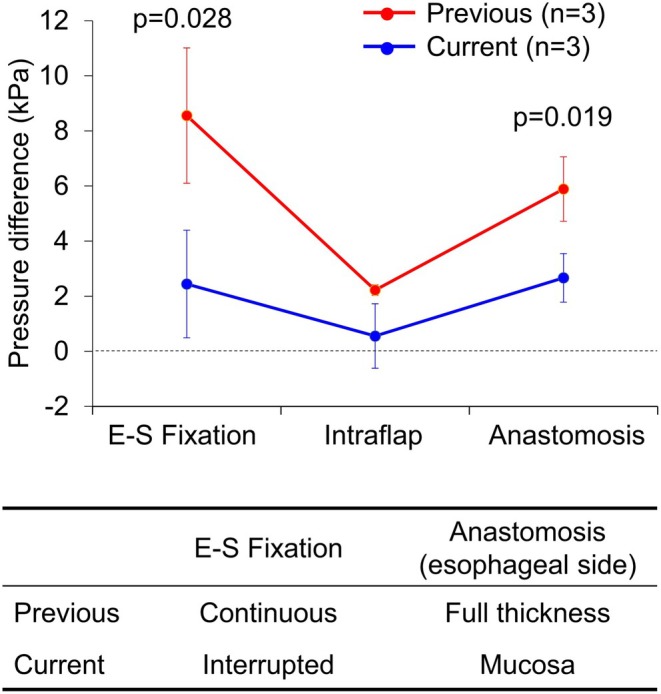
Intraluminal pressure analysis using pig organs. The *Y*‐axis represents the pressure difference between each site indicated on the *X*‐axis and the baseline (the proximal side of the anastomosis).

Finally, intraluminal pressure analysis using the pig organs demonstrated that intraluminal pressure, which was fundamentally higher at the anastomosis and the E‐S fixation site compared with the intra‐flap area, was significantly higher in the previous procedure than in the current procedure, both at the anastomotic site (*p* = 0.019) and at the E‐S fixation site (*p* = 0.028) (Figure 3). This finding indicates that not only the anastomotic site, but also the E‐S fixation site may be a site of obstructive symptoms, and that continuous suturing for E‐S fixation may increase the risk for obstructive symptoms, supporting the analysis in Table 5 based on the clinical data. Technical modification of the suturing procedure on the posterior esophagus at the anastomosis (changing from full‐thickness to mucosal suturing) (Figure 1) resulted in a significant reduction in intraluminal pressure (*p* = 0.019), whereas no significant benefits were observed in the analyses based on the clinical data regarding anastomotic stricture and obstructive symptoms.

## 4 Discussion

Though anastomotic stricture has been widely recognized as the most critical complication in DFT reconstruction, the detailed risk factors and preventive methods for it are still not well understood. Although excessive flap tightness could potentially contribute to anastomotic stricture or obstructive symptoms, there are currently no studies supporting this possibility. The present study showed that mucosal detachment of the esophagus during the anastomosis, defined as insufficient mucosal anastomosis for more than one‐quarter of the circumference, is an independent risk factor for anastomotic stricture, which is a novel finding. Considering the condition after endoscopic submucosal dissection, it can be easily understood that the area where the mucosa has detached will subsequently develop scar contraction, which could potentially lead to stricture. In addition, this study showed that thermal damage to the esophageal mucosa is a significant cause of mucosal detachment of the esophagus, but it was not, on its own, a significant risk factor for anastomotic stricture. Nakao et al. reported in their previous study the importance of preserving the esophageal and gastric mucosa from thermal damage and direct injury to the vasculature to promote wound healing and prevent anastomotic stricture [8], which supports the present findings. To minimize thermal damage to the esophageal mucosa, the selection and appropriate use of the energy device during transection of the esophageal staple line are also important. Based on the findings of the present study, the use of USADs is preferable for esophageal transection. However, when USADs are not used in robotic surgery, bipolar devices, known for their limited thermal spread, may be a suitable alternative. When using MS, it is essential to adjust the output settings, minimize the activation time, and carefully follow the tissue planes to avoid excessive thermal damage to the esophageal mucosa.

Mucosal suturing of the esophagus on the posterior side of the anastomosis, which is a modification from the original procedure, was performed in only 12 of 77 cases (16%) in the present study. Although none of these 12 cases developed anastomotic stricture, no significant difference was observed due to the small sample size. For the same reason, mucosal suturing of the esophagus was not identified as a significant factor for mucosal detachment, despite the absence of mucosal detachment in all 12 cases. Nevertheless, this technical modification may help reduce the risk of mucosal detachment and potentially contribute to preventing anastomotic stricture. As a potential reason for it, in the original procedure of full‐thickness suturing, there was a risk that the mucosal suturing might be neglected due to the focus on suturing the muscular layer of the esophagus out of a desire to prevent anastomotic leakage. However, by focusing solely on mucosal suturing, it is possible to perform the mucosal suturing more accurately, which may help prevent mucosal detachment. In fact, in the present study, full‐thickness suturing was performed in all 5 cases with mucosal detachment. Regarding suturing on the anterior side of the anastomosis, even in the original method, the anterior side is sutured in two layers and is performed under a generally clearer surgical view than the posterior side, which facilitates more secure mucosal suturing. Therefore, stricture due to mucosal detachment is considered less likely to occur on the anterior side than on the posterior side, provided that excessive thermal damage to the esophageal mucosa is avoided. In fact, in the present study, insufficient mucosal suturing on the anterior side that could lead to mucosal detachment was less frequently observed. This modification on the posterior side of the anastomosis may raise concerns about an increased risk of anastomotic leakage. However, considering that the muscle layer of the esophagus and the seromuscular layer of the remnant stomach are fixed at the height of the upper edge of the flap, it can be assumed that mucosal suturing alone is sufficient for the anastomosis (Figure 1). In fact, no anastomotic leakage was observed in this study, despite the limited number of 12 cases in which mucosal suturing was performed.

Regarding the obstructive symptoms 1 month after surgery, continuous suturing at the E‐S fixation site was a potential risk factor, suggesting that interrupted suturing is a preferred technique at the E‐S fixation site for reducing obstructive symptoms. This finding is supported by the intraluminal pressure analysis using pig organs, which showed that the intraluminal pressure at the E‐S fixation site was significantly higher with continuous suturing than with interrupted suturing, and with continuous suturing, it may even exceed the pressure at the anastomotic site. This is consistent with a previous study reporting that anastomotic compliance was lower with continuous suturing than with interrupted suturing [18]. The finding that not only the anastomotic site, but also the E‐S fixation site can be a location for the occurrence of obstructive symptoms in DFT reconstruction is novel and is expected to serve as an important recommendation for preventing deterioration of patients' postoperative quality of life (QOL). Considering that continuous suturing for the second layer on the anterior side of the anastomosis significantly increased the risk of obstructive symptoms on univariate analysis, although no significant increase was found on multivariate analysis, interrupted suturing may be preferred to reduce the obstructive symptoms after DFT reconstruction, as supported by a previous report [19].

Though this study demonstrated several informative findings in DFT reconstruction, there were several limitations. First, this was a single‐center, single‐arm, retrospective study with a limited number of cases, conducted over as long as approximately 8 years. The incidence of anastomotic stricture at our institution was higher than that reported in two multicenter studies (rD‐FLAP Study and lD‐FLAP Study), in which our institution also participated. Although standardized procedures were performed consistently by the surgical team, from the primary surgeon to the assistant, there was still a significant difference in the incidence of anastomotic stricture among individual surgeons (*p* = 0.0166). Therefore, to determine whether the surgical technique‐related factors identified in this study are truly generalizable across different surgeons and institutions, further investigation by multicenter studies is warranted. Second, the collection of information regarding the obstructive symptoms was quite ambiguous, as their severity varied depending on the time period of the study and the attending physician. The use of a standardized questionnaire, such as PGSAS‐45, would have been desirable to reduce these biases. Third, this study focused exclusively on upper GC and did not include cases of EGJ cancer; therefore, the findings were basically derived from intra‐abdominal reconstruction. However, since the reconstruction techniques are also applicable to mediastinal reconstruction, whether through a transhiatal or transthoracic approach, the findings of this study are considered to be applicable to cases of EGJ cancer as well. Fourth, although the robotic approach was used in only seven cases (9%) in this study, it has attracted considerable interest due to its potential to enable more precise suturing than the laparoscopic approach. Its utility in DFT reconstruction will be further evaluated in future studies. Fifth, although mucosal suturing of the esophagus on the posterior side of the anastomosis may represent a meaningful technical modification to reduce mucosal detachment and prevent anastomotic stricture, no significant differences were observed in the present study, likely due to the small sample size. Further investigation with a larger sample size is warranted to validate this potential benefit.

This study demonstrated the importance of avoiding mucosal detachment of the esophagus, which often results from thermal damage, to prevent anastomotic stricture in DFT reconstruction. Further, the preferability of interrupted suturing at the E‐S fixation site to prevent obstructive symptoms after surgery was demonstrated. The widespread recognition of these findings is expected to reduce the incidence of anastomotic stricture, the greatest concern in DFT reconstruction, and contribute to improving patients' postoperative dietary intake and, thus, QOL.

## Author Contributions

**Shinji Kuroda:** conceptualization, methodology, data curation, investigation, formal analysis, visualization, writing – original draft, writing – review and editing. **Yoshihiko Kakiuchi:** data curation, conceptualization, methodology, writing – review and editing. **Satoru Kikuchi:** data curation, writing – review and editing. **Hajime Kashima:** data curation, writing – review and editing. **Nobuhiko Kanaya:** data curation, writing – review and editing. **Shunya Hanzawa:** data curation, writing – review and editing. **Kenjiro Kumano:** visualization, writing – review and editing. **Masahiko Nishizaki:** supervision, writing – review and editing. **Shunsuke Kagawa:** supervision, writing – review and editing. **Toshiyoshi Fujiwara:** project administration, supervision, writing – review and editing.

## Ethics Statement

This study conformed to the provisions of the Declaration of Helsinki, and the protocol was approved by the Okayama University Hospital Institutional Review Board (Approval no. 2504‐027).

## Conflicts of Interest

The authors declare no conflicts of interest.

## Supporting information


**Video S1:** Intraluminal pressure measurement.
